# Supplementary material for: Content-rich biological network constructed by mining PubMed abstracts
Source: BMC Bioinformatics. 2004 Oct 8;5:147. doi: 10.1186/1471-2105-5-147 (PMC528731; doi:10.1186/1471-2105-5-147)
Supplement: Additional File 2 — The original results of the above study (non-essential files are deleted to keep the file size under the limit set by BMC bioinformatics). [file 1471-2105-5-147-S2.bz2 › chilibotAdditionalFile2/dip05/310ID10369872E1339/html/PSEN1_RAB11A.html]

 


 **PSEN1** and **RAB11A** 
  
Found 3 abstracts in PubMed, retrieved 3.  
 

 What does Google say? 
 PDF only 
| .edu only 

---

**Interactive relationship** (e.g. stimulation, inhibition, etc)

**Neutral relationship**- This suggested that Rab11  [ **RAB11A** ]  presenilin  [ **PSEN1** ]  interaction could influence the routing of betaAPP and thereby modulate its maturation.  Ref: 10973821 Biochem Biophys Res Commun, 2000
- We report an interaction between the human PS1  [ **PSEN1** ]  or PS2 hydrophilic loop and Rab11  [ **RAB11A** ] , a small GTPase belonging to the Ras related superfamily.  Ref: 10369872 Hum Mol Genet, 1999
- Interaction domains were mapped to codons 374 400 for PS1  [ **PSEN1** ]  and to codons 106 179 for Rab11  [ **RAB11A** ] , a region including the fourth GTP binding domain.  Ref: 10369872 Hum Mol Genet, 1999

**Non-interactive relationship** (e.g. studied together, co-existance, homology, etc.)

- However, it does not preclude the possibility for Rab11  [ **RAB11A** ]  to modulate other presenilin  [ **PSEN1** ]  mediated functions in human cells.  Ref: 10973821 Biochem Biophys Res Commun, 2000
